# Salvage hepatectomy in refractory pyogenic liver abscess: a case report

**DOI:** 10.1093/jscr/rjaf891

**Published:** 2025-11-14

**Authors:** Sophia Chan

**Affiliations:** Department of General Surgery, Cairns Hospital, 165 The Esplanade, QLD 4870, Australia

**Keywords:** pyogenic liver abscess, hepatectomy, percutaneous drainage failure

## Abstract

Pyogenic liver abscess (PLA) is a rare but life-threatening condition. First-line management includes antimicrobial therapy and image-guided percutaneous drainage. Surgical resection is rarely required but remains an important method for definitive source control in selected cases. We report the case of a 65-year-old man with a large hepatic abscess that remained refractory to percutaneous drainage. Culture of the drain aspirate grew *Klebsiella aerogenes* and *Candida albicans*. Despite appropriate antimicrobial therapy, the patient’s sepsis persisted, and imaging demonstrated inadequate resolution of the abscess. The patient underwent a hepatectomy and cholecystectomy. The post-operative course was complicated by a prolonged ileus, and the patient was well in his outpatient follow-up a few months after discharge. While uncommon in the modern era, hepatectomy remains a definitive treatment option for hepatic abscess when percutaneous drainage fails. Early recognition of treatment failure and timely escalation to surgery can be lifesaving.

## Introduction

Pyogenic liver abscess (PLA) can be a life-threatening, albeit uncommon, cause for intra-abdominal infection. The incidence is gradually increasing, with previously described male predominance and a higher affinity to those between the ages of 65 and 85 years old [1]. Etiology can be categorized based on the causative organism or associated disease, or procedure. The most common organisms described in the literature include the *Streptococcus* species, *Escherichia, Staphylococcus,* and *Klebsiella* [2]. Diseases associated with PLA include biliary disease, intra-abdominal collections, and bile duct ischemia secondary to liver transplant, interventional procedures, trauma, or surgical instrumentation of pancreas or duodenum. Contamination can occur with biliary infection, direct extension, hepatic arterial seeding, portal vein seeding, penetrating trauma, immunosuppression, iatrogenic, or cryptogenic causes [1].

Over time, mortality and morbidity rates have reduced due to improvements in diagnostic imaging, antimicrobial therapy, and minimally invasive interventional radiology [3]. Current first-line management for PLA involves intravenous antibiotics with imaging-guided percutaneous drainage where applicable [3]. Indications for surgical intervention include failed percutaneous drainage, large septated or multiloculated collections, lesions in unfavorable anatomical locations, rare pathogens (*Klebsiella* hypervirulent strains and parasitic infections), iatrogenic complications, or underlying complex pathology (i.e. associated malignancy) [4]. In particular, *Klebsiella*-related liver abscesses can cause aggressive, multiloculated abscesses that are refractory to percutaneous drainage [5].

We describe the case of a man with a large multiloculated hepatic abscess that was refractory to percutaneous drainage who subsequently underwent surgical intervention. This case report highlights the challenges that arise where management may deviate from first-line therapy, operative considerations, and the role of surgical resection in the modern management of PLA.

## Case presentation

A 65-year-old Indigenous male from Weipa presented with a 3–4 week history of abdominal pain, poor oral intake, fevers, and diarrhea. The patient had a history of hypertension and dyslipidemia for which he was medicated, and no previous abdominal surgeries. Notable social history included heavy alcohol, tobacco, and marijuana use.

On arrival at the hospital, the patient was hemodynamically stable, and all vitals were within normal limits. Mild right upper quadrant and epigastric tenderness were noted; however, there was no peritonism. Laboratory investigations revealed marked leukocytosis (WCC 27.1), C-reactive protein level of 315, elevated liver function tests (ALP 374, GGT 267, AST 252, ALT 148), hyperbilirubinemia (Bili 30), and a normal lipase. Blood cultures grew gram-negative bacilli and gram-positive cocci.

**Figure 1:**
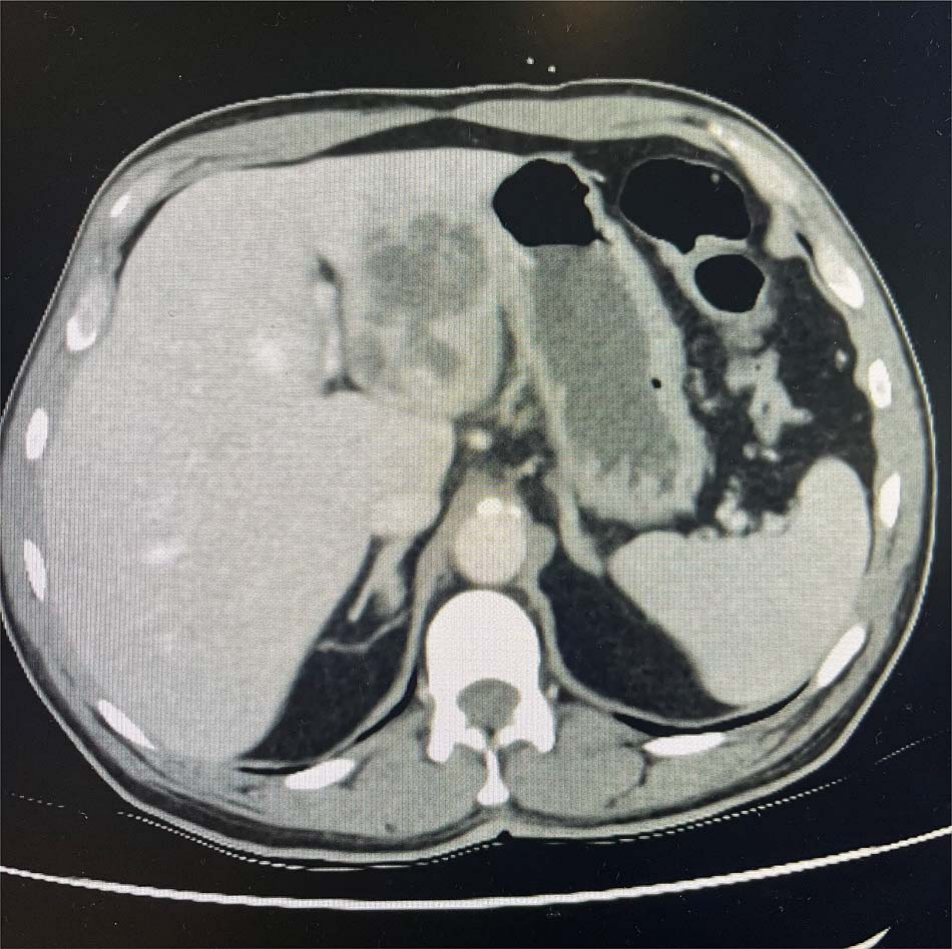
CT abdomen and pelvis in portal venous phase in axial slice. Hepatic abscess seen in left hepatic lobe.

**Figure 2:**
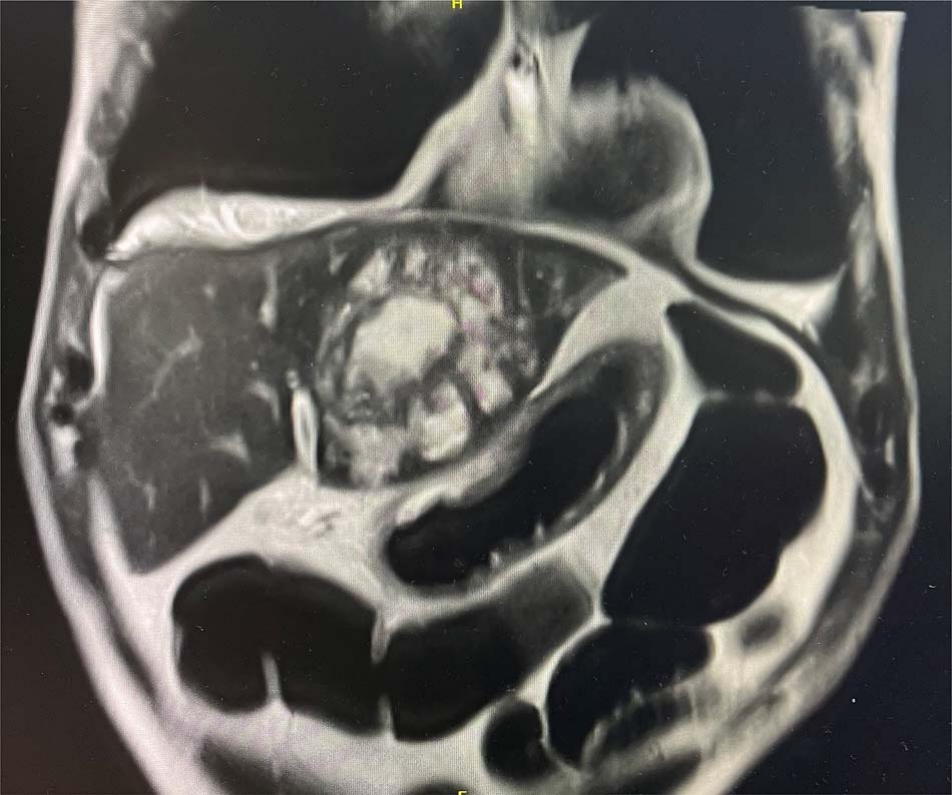
MRI liver in coronal slice. Hepatic abscess seen in left hepatic lobe.

Cross-sectional imaging with ultrasound, computed tomography (CT) (Fig. 1), and magnetic resonance imaging (MRI) (Fig. 2) showed a rapidly-growing large, complex lesion measuring ~55 × 39 × 43 mm in the left hepatic lobe, with features suggestive of an evolving abscess but no drainable collection. Empiric IV piperacillin-tazobactam was commenced.

Shortly after admission to the ward, the patient developed septic shock and new-onset rapid atrial fibrillation, prompting an admission to the Intensive Care Unit for vasopressor support and escalation of intravenous antibiotics to Meropenem. Imaging-guided percutaneous drainage was attempted; however, no purulent material was aspirated. A second opinion was obtained from the Gastroenterology team, and differentials included a hydatid cyst or an amoebic abscess, along with a recommendation for an endoscopic ultrasound (EUS). Empirical treatment for a hydatid cyst was commenced, and the patient was given hydrocortisone and albendazole.

The EUS demonstrated mixed solid and cystic characteristics of the lesion, and culture of the fluid aspirated grew *Klebsiella aerogenes* and *Candida albicans*. Antimicrobial therapy was tailored to include intravenous cefepime and oral fluconazole. The patient was stepped down from the ICU after weaning off vasopressor support and resolution of rapid atrial fibrillation. Over the course of a few days, the drain output was consistently hemoserous and under 100 mL in output; inflammatory markers remained markedly elevated.

Ongoing fevers and a repeat CT after 1 week showed a stable appearance of the multi-loculated liver abscess; however, a mispositioned drain and new perihepatic and perisplenic collections. The drain was repositioned, and hemobilious output followed. Another repeat CT completed a further week later showed minimal improvement, and the decision was made to proceed with a left hepatectomy, cholecystectomy, and intraoperative cholangiogram. Intraoperative findings report a firm mass with multiple foci of liver abscesses with no discrete abscess and likely biliary obstruction secondary to mass effect. The intraoperative cholangiogram was very difficult due to a heavily fibrosed cystic duct, warranting dissection down to the common bile duct. Generous lavage with three liters was conducted, and a drain was left *in situ*. Histopathology confirmed a necrotic liver abscess without evidence of malignancy or hydatid disease. Inflammatory markers improved significantly post-operatively.

Postoperatively, the patient developed an ileus and required temporary total parenteral nutrition (TPN). There was a slow resolution of an ileus, and the patient was discharged thirty days later with oral antibiotics. He remained well at his 6-month outpatient follow-up.

## Discussion

The management of PLA has evolved significantly with time. Historically, open surgical drainage was the mainstay treatment; however, there has been a shift toward less invasive procedures such as percutaneous drainage, and this is heavily supported by the literature. Despite this, there remains a small group of patients who fail to respond, necessitating escalation to operative intervention.

A literature review has revealed ~12 cases that involved surgical intervention due to failed percutaneous drainage, with improved survival attributed to definitive source control. In our case, the patient had ongoing sepsis and minimal radiological change despite two percutaneous drainage attempts. The anatomical location of the abscess was deemed amenable to hepatectomy, and the decision was made to proceed with surgical intervention to gain source control. This case underscores that, while rarely indicated, hepatectomy remains a valid and potentially life-saving option in modern PLA management when less invasive measures fail.

## Conclusion

Although percutaneous drainage remains the cornerstone of PLA management, a small proportion of cases will fail to respond and require surgical intervention. Hepatectomies can provide a definitive option when source control cannot be achieved by less invasive means. This case highlights the value of timely recognition of drainage failure and the role of surgical intervention in achieving definitive treatment and source control in selected patients.
